# Visualising accelerometer-based 24/7 human movement behaviour data: an umbrella review and framework development from the LABDA project

**DOI:** 10.1186/s44167-025-00088-6

**Published:** 2025-11-18

**Authors:** Marian Marchiori, Josef Heidler, Gaia Segantin, Henrik R. Eckmann, Mai J. M. Chinapaw, Morten Kjærgaard, Jasper Schipperijn

**Affiliations:** 1 Department of Sports Science and Clinical Biomechanics, Faculty of Health, University of Southern Denmark, Odense, Denmark; 2 Amsterdam University Medical Center (UMC), Public and Occupational Health, Amsterdam, The Netherlands; 3 Amsterdam Public Health, Health Behaviors & Chronic Diseases and Methodology, Amsterdam, The Netherlands; 4 Diabetes Research Centre, Leicester General Hospital, University of Leicester, Leicester, UK; 5 Leicester Biomedical Research Centre (BRC), National Institute for Health Research (NIHR), University Hospitals of Leicester NHS Trust, University of Leicester, Leicester, UK; 6 SENS Innovation ApS, Copenhagen, Denmark

**Keywords:** Wearables, 24/7 movement behaviours, Physical activity, Sedentary behavior, Sleep, Communication, Visualisation methods

## Abstract

**Background:**

Regular Physical Activity (PA) is important for disease prevention and health promotion. PA has been assessed through surveys, questionnaires, and devices such as accelerometers. Alongside PA, Sedentary Behaviour (SB) and sleep are the main components of 24/7 movement behaviours, and their adequate measurement is important for assessing health outcomes. Many different metrics to summarise 24/7 movement behaviours are used; however, little attention has been paid to visualising these metrics. Data visualisation is likely to impact the way results are communicated and understood by different audiences. This study systematically reviews 24/7 movement behaviour metrics, presents an overview of their visualisations, and develops a framework to guide context-specific visualisation choices.

**Methods:**

An umbrella review was conducted in February 2025 in Scopus and Web of Science. Included papers were reviews of any type, with any human population and study design, having at least one of the three 24/7 movement behaviours as exposure or outcome measured through accelerometers, and clearly reporting the outcome metrics. Data extraction and an adapted thematic data analysis were performed in April 2025. The overview of the visualisations used for the metrics identified in the review and thematic analysis was created through non-systematic web searches and use of Microsoft Copilot. Finally, a framework was created based on the sender-receiver model for effective communication.

**Results:**

In total, 93 reviews were included, with a total of 5667 articles reporting on 134 unique output metrics based on accelerometer data. The most common metrics were step counts and time spent in Moderate-to-Vigorous Physical Activity (MVPA). The non-systematic web searches showed that most researchers use bar charts, line graphs, or pie graphs to visualise 24/7 movement behaviour data, while Copilot input provided more options of visualisations. The resulting framework was the product of an iterative process aggregating the previous results, providing clear guidance for organising metrics and their corresponding visualisations.

**Conclusions:**

This study structures and summarises types of visualisations of accelerometer-derived metrics to describe 24/7 human movement behaviour data. Future research is needed to apply the framework in practical contexts and investigate how the visualisations are perceived by different audiences.

**Supplementary Information:**

The online version contains supplementary material available at 10.1186/s44167-025-00088-6.

## Background

Regular Physical Activity (PA) is important for disease prevention and health promotion and has a positive effect on many different health outcomes, such as cardiovascular diseases, cancer and diabetes [1]. To promote adequate PA according to the current World Health Organization (WHO) guidelines [2], monitoring population PA levels is crucial. However, measuring PA is challenging because it has multiple dimensions [3], including Frequency, Intensity, Time, Type (FITT) [4], and can therefore be described in multiple ways, using various metrics.

Alongside PA, Sedentary behaviour (SB) and sleep are the main components of 24/7 human movement behaviours [5–7]. SB, characterised by low-energy activities such as sitting, and sleep, essential for physiological recovery, have both been associated to health outcomes [8–11]. Over the past decade, an increasing number of studies have emphasised the integrated health benefits of high PA, low SB, and adequate sleep across the lifespan [12], which aligns well with the most recent public health guidelines [2]. Therefore, the adequate measurement of 24/7 movement behaviours is important when looking at the relation with health outcomes.

Historically, 24/7 movement behaviours, with focus on PA, have been assessed through surveys and questionnaires, such as the International Physical Activity Questionnaire (IPAQ) [13] and Global Physical Activity Questionnaire (GPAQ) [14]. Technological advancements have led to the increased use of wearable sensor techniques such as accelerometers, providing rich time-series data over longer periods [15]. However, because 24/7 movement behaviours are complex and cannot be represented by a single measure, as height and heart rate can, it is hard to capture all their dimensions with questionnaires or accelerometers [16]. The different methods for measuring 24/7 movement behaviours, summarised by Sylvia et al. [17], give us different output metrics. Adding further complexity, the most commonly used metrics, such as accelerometer counts, “lack meaning in clinical and public health settings”, as said by Rowlands et al. [18].

In summary, one of the main challenges in working with 24/7 movement behaviour data is that there is no one-size-fits-all approach. Researchers inevitably need to make choices regarding which behavioural dimensions to assess and which metrics to use, depending on the research focus, objectives, and populations [17]. Making these choices explicit, transparent, and guided by clear rationale would not only enhance the communication of the intended message to different target groups, but also promote a more consistent and informed use of 24/7 movement behaviour metrics across studies. Supporting researchers in navigating these decisions more easily and transparently is a key step toward advancing the field. As part of this effort, the EC-funded Learning Network for Advanced Behavioural Data Analysis (LABDA) consortium aims to advance methodologies, improve interpretability, and provide tools to help researchers address these challenges, and the present work contributes to this collective initiative.

A direct, clear, and easy way of communicating research results is through data visualisation [19]. Data-visualisation is likely to impact the way results are understood, and optimal ways of visualising results may vary across audiences, including researchers from different fields [20]. However, there are many different ways of measuring 24/7 movement behaviours, resulting in many different output metrics, which can be visualised in many different forms. Thus, beyond measuring 24/7 movement behaviours, the way the resulting data and metrics are visualised, communicated, and transformed into meaningful messages for different audiences may be equally, if not more, important [21], so they can be used for data-driven behavioural change policy making as well as individual behaviour change, for instance when visualisations are used as feedback in interventions.

Despite the increasing availability of accelerometer data, little attention has been paid to how best to visualise and communicate 24/7 movement behaviour data. Researchers still lack evidence on which visualisations are most easily understood by different audiences, depending on the output metric, research question, and other study-specific factors. As a result, the choice of visualisations might be guided mainly by the characteristics of the data, and the message researchers wish to convey, rather than by explicit consideration of the preferences or needs of different audiences. Consequently, the communication purpose may not always be fully addressed, which hinder the effective transfer of knowledge to various audiences, such as policy makers, health professionals, and end users of wearable technology [22]. To address this issue, we aimed to develop a communication framework that explicitly considers how the information is coded by the sender in a visual way. In this study, we adopt the sender-receiver model for effective communication [23] to support researchers in choosing visualisations that align not only with the characteristics of the data but also with the needs and expectations of the target audience. By providing an overview of accelerometer metrics and their respective visualisations, along with guidance on when to use them, we aim to reduce confusion, enhance transparency, and improve the effectiveness of communication in movement behaviour research.

With that in mind, the present study has the main aim of finding out which types of visualisations are more suited for each output metric considering the research context, in order to help movement behaviour researchers better communicate their results. To achieve this aim, we:

1. Conducted an umbrella review of metrics used to describe 24/7 movement behaviour accelerometer data.
2. Created an overview of visualisations used for these metrics.
3. Developed a framework connecting the findings from the umbrella review and visualisations overview with existing literature, adding the research context to the process of choosing the most appropriate data visualisations.

## Methods

The first step was to perform an umbrella review to identify the accelerometer-derived metrics used to describe 24/7 human movement behaviour. This umbrella review followed the methodology of a scoping review; therefore, the reporting was guided by the standards of the Preferred Reporting Items for Systematic Reviews and Meta-Analyses for Scoping Reviews (PRISMA-ScR) [24], and documented through the PRISMA-ScR checklist ( Supplementary material1). No protocol was registered for this review. For each metric (or group of metrics), we then summarised the visualisations identified through our overview as applicable for communicating them. Based on the literature review and the overview of visualisations, we developed a framework to guide researchers towards suitable visualisation methods for different types of typical research questions regarding 24/7 movement behaviour accelerometer data.

### Umbrella review

#### Search strategy

The literature search was conducted on February 10th, 2025, both in Scopus, which includes MEDLINE and EMBASE, and Web of Science, using the same search block ((“movement behavio*” OR “activity behavio*” OR “exercise” OR “physical activity” OR “sedentary behavio*” OR “sleep”) AND (“accelerometer-measured physical activity” OR “accelerometer data” OR “wearable electronic devices”) AND (metric* OR variable* OR measure* OR outcome* OR output*)) applied to “Title, abstract, and full text” for Scopus, and “All fields” for Web of Science. The search strategy was developed by the authors, informed by relevant terminology, operational definitions, and Medical Subject Headings (MeSH) terms. To further refine the search, we applied the following filters: publication year from 2010 to January 2025; published in English; and reviews limited to the final stage of publication according to the Scopus criteria, which includes all types of reviews and meta-analyses. The start date of 2010 was selected to capture the most recent 15 years of research, reflecting contemporary approaches to measuring 24/7 movement behaviours, while the umbrella review design ensured that earlier studies were still captured through the included reviews.

#### Inclusion and exclusion criteria

The included papers were reviews of any type, with any human population, including any design of quantitative studies or a mix of quantitative and qualitative studies. They had at least one or any combination of the three 24/7 movement behaviours (PA, SB, and sleep) as either exposure or outcome, with at least one study included based on accelerometer data, and should report a summary of the outcome metrics clearly in the review text or supplementary material. The 24/7 movement behaviours did not need to be the only exposure/outcome considered; they could be combined with any other measures of interest. Protocols for reviews were excluded, as well as papers not written in English, and papers with no full text available.

#### Review selection

Title and abstract screening were done in two independent rounds. Initially, the first author (MM) performed screening of all reviews found, and in a second round, three other authors (JS, GS, and JH) shared the screening of the reviews, so that each review was independently screened by two authors. When there was disagreement, the authors discussed and tried to reach a consensus. If no consensus was reached, a fourth author (MK) was invited to resolve conflicts. Full text screening followed the same two rounds and conflict resolution procedure. The Covidence systematic review software (Veritas Health Innovation, Melbourne, Australia) was used to handle duplicates, so no manual removal was needed, and to streamline the workflow during screening and data extraction.

#### Data extraction

For each of the reviews included we extracted information on title, first author, review publication year, journal, DOI, type of review, range of publication year of included papers, study design of included papers, number of papers included, number of papers which used accelerometers as an assessment tool, population characteristics (age and disease status), exposure(s), outcome(s), if visualisation tools were discussed or mentioned, and the accelerometer-derived output metrics used to describe 24/7 movement behaviours. Data extraction was performed by the first author (MM) using a pre-defined form built within Covidence, which reflects the structure of Supplementary Material 2. The form was piloted on five reviews and refined before being applied to all included reviews. There was no missing data. The resulting data was exported to a spreadsheet ( Supplementary Material 2).

#### Synthesis of results: thematic analysis of accelerometer metrics

An adapted thematic data analysis [25] was performed by MM, in which all the output metrics compiled in the data extraction phase were recorded in a spreadsheet until no new metrics appeared. Repeated metrics were not recorded, and variations of the same metric were phrased in the simplest encountered form, to streamline the coding process and ensure consistency in how themes were identified and described [26]. General metrics such as “activity types” with no reference to specific categories were not included. The first author (MM) then grouped the metrics into themes that emerged from this analysis. This process was conducted as a reflexive and interpretive exercise rather than a strictly prescriptive one, with framework categories being formed iteratively from the metrics and the data they represent, and refined against existing literature terminologies [27].

For this review, we compiled only output metrics that can be generated by a single accelerometer through any processing method, including machine learning algorithms, which represent human movement behaviour [28], as opposed to input or summary metrics, which quantify the magnitude of acceleration and are used to derive output metrics [29]. Examples of summary metrics include Euclidean Norm Minus One (ENMO), Monitor-Independent Movement Summary (MIMS) units, and Activity Intensity (AI0). We did not assess the validity or accuracy of the methods used to derive the output metrics from accelerometry.

### Overview of visualisations

To create an overview of the visualisations used for each metric or group of metrics identified in the thematic analysis, we first tried to perform systematic searches in PubMed (which draws from the EMBASE database). We focused our search for visualisations on PubMed, as results from Scopus largely overlapped and Web of Science yielded many irrelevant records, allowing efficient and relevant coverage of the considerable number of metrics. The search blocks used to attempt this search are presented in detail in the Supplementary Material 3.

These searches returned studies measuring behaviours with accelerometers, with visualisations included for illustration of results rather than as a focus of systematic investigation, which was our objective. Since the results were not useful to answer our research questions, we performed non-systematic web searches using Google and inputting the same model of search blocks as described above. The searches were conducted by the first author in May 2025. This stage was not part of the systematic umbrella review but rather a separate, exploratory process aimed at compiling visualisations to map onto the metrics and support the subsequent development of the framework.

Additionally, Microsoft Copilot Pro, a web-based generative artificial intelligence tool, was used. We first asked Copilot about the most common visualisations used for each group of metrics with the prompt: “Can you please name and describe the most common visualisations, such as graphs, plots, charts and similar tools, used to communicate (specific metric or group of metrics) derived from accelerometer data assessing 24/7 movement behaviour data?”. This was done for all groups of metrics compiled, and when necessary, for specific metrics as well. We furthermore asked Copilot, in a new session to get independent answers, to identify the most common uses of each visualisation. The prompt used was “Can I use (list of visualisations given by Copilot in a previous conversation) to correctly communicate (specific metric or group of metrics) derived from accelerometers to communicate 24/7 movement behaviour data?”. We also made a version of these prompts for each one of the movement behaviours (PA, SB and sleep), but the results were redundant.

### Framework development

**Fig. 1:**
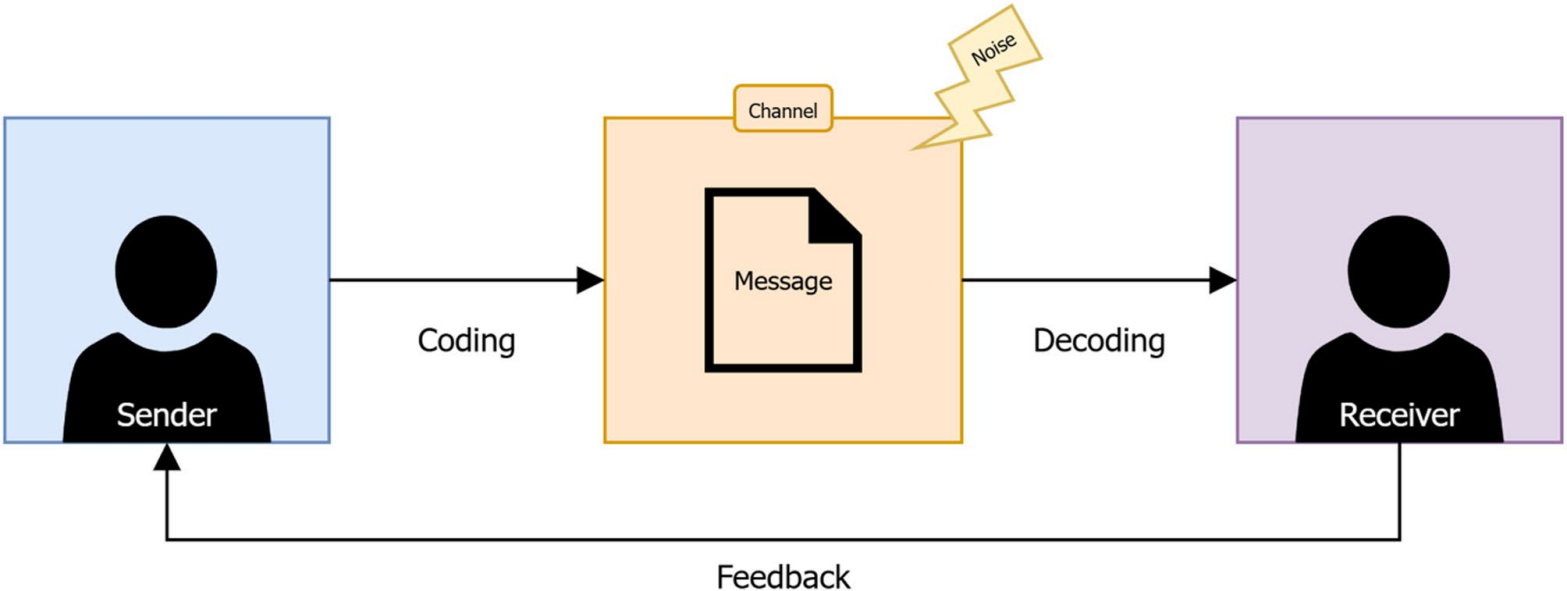
The sender receiver model for effective communication, as described by Planer and Godfrey-Smith [23]

To guide researchers in selecting suitable visualisations for the accelerometer metrics of their choosing, a framework was developed, informed by both existing literature [20, 22] and the results from the umbrella review and non-systematic visualisations search. The rationale for the framework was based on the sender-receiver model for effective communication [23]. This model explains how information is exchanged between two parties, the sender (who transmits the message) and the receiver (who receives and interprets the message). It also outlines key components of the communication process, including message, coding process, channel, decoding process, feedback, and noise. The model is visually summarised in Fig. 1.

The framework was designed to help researchers achieve clear communication of their message, trying to improve the coding step of the communication, ultimately enhancing the impact of their research. How well the message is transmitted to different target audiences considering visualisation characteristics and other noises, and how the message is interpreted, or decoded, on the other side of the model, will be focus of future research.

## Results

### Umbrella review

**Fig. 2:**
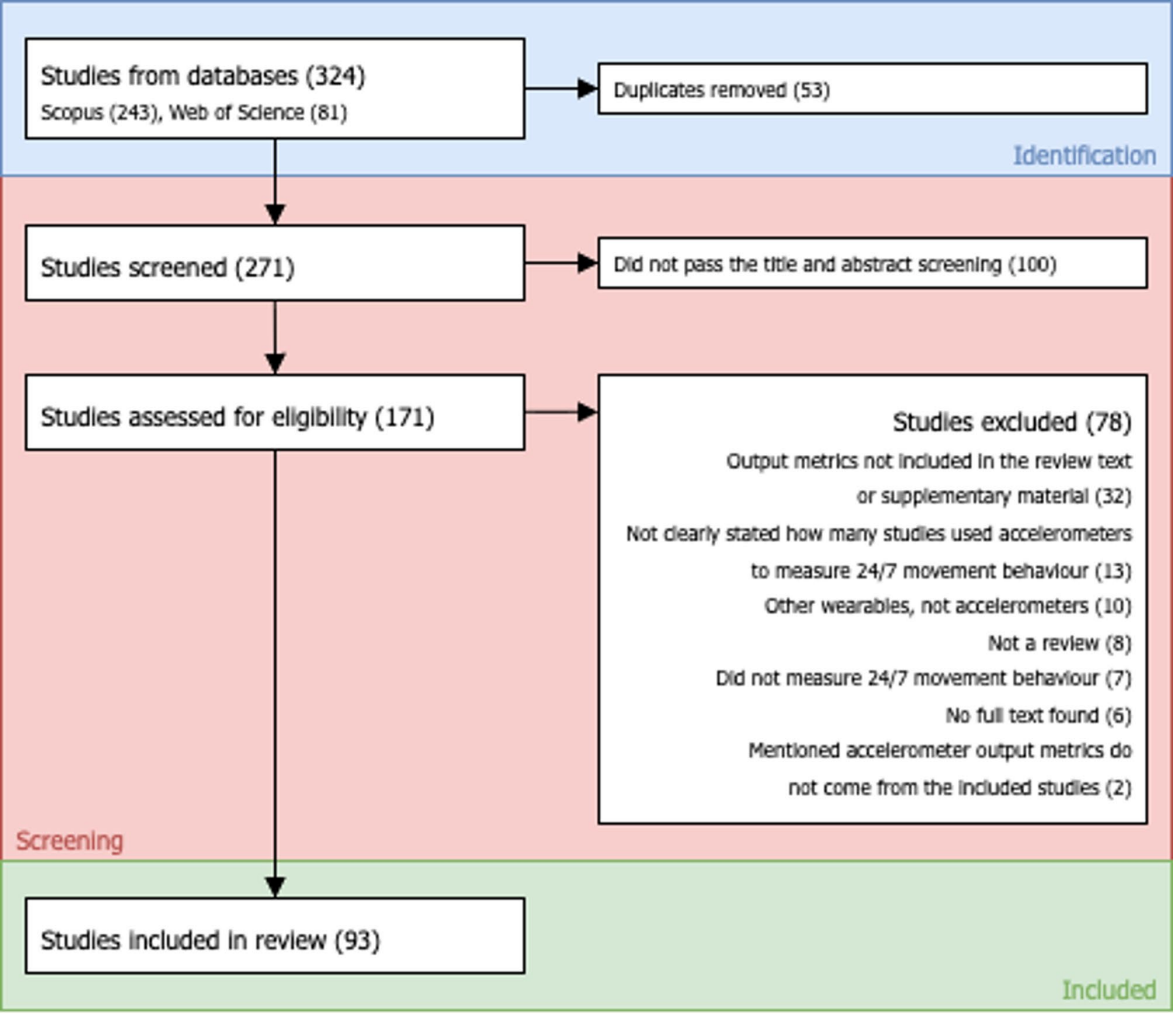
Adapted PRISMA flow chart of the process of identification, screening, and selection of reviews

A total of 324 reviews was found. After duplicates were removed, 271 reviews were assessed for eligibility, and 171 reviews met the inclusion criteria after screening of title and abstract. The full texts of 171 reviews were assessed, and 93 reviews were included. Figure 2 presents the flow chart for the umbrella review, adapted from PRISMA [30].

The 93 included reviews [31–123] were published between 2012 and 2024. The papers included in the reviews were published between 1985 and 2023, with most of the reviews (*n* = 74) containing papers published from 2000 and onwards. Of the 93 reviews, 53 were systematic reviews, 14 were systematic reviews and meta-analyses, 21 were scoping reviews, 4 were narrative reviews, and 1 was an umbrella review. Altogether, the 93 reviews included a total of 5,667 papers, not accounting for duplication, of which 4,669 relied on accelerometers to measure one or more 24/7 movement behaviours. The reviews included different types of studies, across different populations, and in different fields of research, providing a comprehensive foundation for the overview of 24/7 movement behaviour metrics. None of the reviews discussed or mentioned visualisation tools for 24/7 movement behaviour data or metrics. The data extracted from the 93 reviews are summarised in Supplementary Material 2.

A total of 134 unique output metrics used to communicate accelerometer data were identified. The most common metrics were step counts, which was cited in 67 out of the 93 reviews, followed by time spent in MVPA (cited by 65 reviews), time spent in Light physical activity (LPA) (cited in 50 reviews), counts, total time spent in SB, and total time spent in PA, cited by 48, 43 and 40 reviews, respectively. Five themes were developed from the thematic analysis: Relative measures, Frequency, Absolute time, Point in time, and Summary/Pattern/Derived Measures. Table 1 provides an overview of metrics used to describe 24/7 movement behaviour data, grouped by these themes, considering how many reviews each metric appeared in. Supplementary Material 4 details the process of coding and thematic data analysis, and better describes the output metrics, including the units of measurement and time granularity (e.g. time in seconds, minutes hours, days, etc.).

**Table 1:** Overview of 134 accelerometer-derived output metrics and how many reviews cited each metric

Relative measures ^a^	Frequency ^b^	Absolute time ^c^	Point in time ^d^	Summary/Pattern/Derived Measures ^e^
Sleep efficiency (9)	Sit-to-stand transitions (12)	MVPA (65)	Wake time (13)	Step count (67)
Relative MVPA (6)	Bouts of PA (11)	LPA (50)	Sleep onset (5)	Counts (48)
Relative SB (5)	Step/walking bouts (9)	Total SB (43)	Bedtime (3)	Estimated energy expenditure (30)
Rest/activity rhythm (r24) (5)	Bouts of MVPA (7)	Total PA (40)	Foot fall (1)	METs (33)
Relative LPA (4)	Lie-to-stand transitions (6)	MPA (38)		Walking distance (17)
Sleep/wake rhythm (4)	Sleep fragmentation (6)	VPA (37)		Walking speed (17)
Nighttime restfulness (I < O) (4)	Bouts of VPA (5)	Walking (31)		Peak performance (7)
Restlessness Index (3)	Meet PA guidelines (5)	Sitting (22)		Stairs climbed (4)
Relative PA (2)	Bouts of SB (4)	Step duration (21)		Stride length (3)
Relative MPA (2)	Bouts of LPA (3)	Standing (17)		Estimated oxygen consumption (2)
Relative VPA (2)	Bouts of MPA (3)	Lying (17)		Estimated loading rate (BWf/s) (2)
Relative VVPA (2)	Sit-to-lie transitions (3)	Running/jogging (10)		Step length (2)
Relative sitting (2)	Active-to-sedentary transition (2)	VVPA (8)		Activity amplitude (1)
Relative lying (2)	High-low activity transitions (2)	Cycling (8)		Social jet lag (1)
PA level (total EE/resting EE) (1)	Sleep bouts (2)	Upstairs/downstairs (7)		Counts in the most active interval (1)
Swing percentage (1)	Arm flexion/extension (2)	Sleep onset latency (7)		Maximum walking speed (1)
A/T ratio (acceleration during the daytime over acceleration during sleep) (1)	Sitting bouts (2)	WASO (7)		Step regularity (1)
Relative walking (1)	Stand-to-walk transitions (1)	Time in bed (5)		Stride regularity (1)
Relative standing (1)		Brisk walking (5)		Vertical displacement (1)
		Mean bout length (4)		Gait (walking) variability (1)
		Driving/riding a vehicle (4)		Stride count (1)
		Turning (4)		Intensity of ambulation activity bouts (1)
		Non-locomotive PA (3)		Number of days/weeks with at least 10 min/day of MVPA (1)
		Upright (3)		
		Wake time in bed (3)		
		Leaning/reaching (3)		
		Jumping (3)		
		Daytime max activity (3)		
		PFWA (2)		
		Aerobic PA (2)		
		Light sleep (2)		
		Deep sleep (2)		
		REM sleep (2)		
		Max bout length (2)		
		Stride duration (2)		
		Arm flexion/extension (2)		
		Sitting bouts (2)		
		Crawling (2)		
		Being carried (2)		
		Double support duration (2)		
		Stance time (2)		
		Up intervals /wakefulness (2)		
		Mean PA during UP interval (2)		
		Stride time (2)		
		VLPA (1)		
		Household activity (1)		
		Longest sleep episode at night (1)		
		Longest wake episode at night (1)		
		Falling (1)		
		Wiggling (1)		
		Rolling (1)		
		Walking on tiptoe (1)		
		Clapping hands (1)		
		Dancing (1)		
		Rowing (1)		
		Strength/weights (1)		
		Garden (1)		
		Throwing (1)		
		Riding a toy (1)		
		Outdoor time per day (1)		
		Step duration (1)		
		Stance duration (1)		
		Swing duration (1)		
		Single support duration (1)		
		Swing time (1)		
		Riding up and down an elevator (1)		
		Time in incidental movement (1)		
		Wheelchair driving (1)		
		Unspecified non-cyclic movement (1)		
		Step time (1)		

^a^Derived from a ratio of two other measures. Most often represents relative time but can also include proportions of other quantities. ^b^ Count of how many times a specific behaviour occurs. ^c^ Total duration of a behaviour. Usually expressed per day, but can also be per bout, minute, hour, week, or other units, depending on the research question. ^d^ Represented by a specific time stamp. ^e^ This category includes derived metrics that reflect variability, stability, or temporal patterns over time. These are not easily classifiable as time-point, duration, frequency, or proportion

Abbreviations: MVPA = Moderate to Vigorous Intensity Physical Activity. SB = Sedentary Behaviour. LPA = Light Intensity Physical Activity. MPA = Moderate Intensity Physical Activity. VPA = Vigorous Intensity Physical Activity. VVPA = Very Vigorous Physical Activity. EE = Energy Expenditure. WASO = Wake after sleep onset. PFWA = Pain free walking ability. REM = Rapid Eye Movement. VLPA = Very Light Physical Activity. MET = Metabolic Equivalent of Task

### Overview of visualisations

Regarding the visualisations, the non-systematic web searches showed that most studies rely on bar charts, line graphs, or pie charts to visualise 24/7 movement behaviour data. However, these visualisations are typically used without a clear rationale for selecting one method over another. Further searches and Copilot input identified a total of 28 different types of visualisations (Additional File 5) applicable to the metrics and metric groups compiled in this review. This indicates that researchers do not use all the possibilities available for the type of data they have.

## Framework development

The results of the umbrella review revealed a lack of guidance on how to visualise accelerometer-derived 24/7 movement behaviour metrics. Rather than following up with a standard discussion, we added this section to present a framework that addresses this gap.

We first examined the characteristics of the data from which these metrics are derived. 24/7 movement behaviour data, like many forms of sensor-based data captured continuously [124], are generally processed in one of two ways: as unclassified data, with specific output metrics (e.g. counts) calculated from raw acceleration [125–127]; or as classified data [128], through methods that generate output metrics representing either types of behaviours/activities (e.g. walking, sitting, running) [129] or intensity levels (e.g. MVPA) [130].

Drawing from the literature [131, 132] and our own review and exploratory searches, we observed that unclassified 24/7 movement behaviour data produce output metrics related to intensity, or quantity of activity, over time. They usually answer research questions related to “How much?” of a behaviour is happening. In contrast, metrics from classified data (either representing types of activity or intensity levels) are commonly used to describe: point in time, duration, proportion, and frequency, though they are found in the literature with different terminologies [28, 133, 134]. Point in Time refers to the exact moment when a behaviour starts or ends, corresponding to the research question “When?”. Duration is the length of time a behaviour occurs, answering the research question “How long?”. Proportion is the relative amount of time spent in a specific behaviour or intensity, addressing “What is the relative importance?”. Frequency is the recurrence or regularity of a given behaviour, reflecting “How often?” it happens.

These features aligned with the thematic categories that emerged during our analysis of the compiled metrics. The “Summary/Pattern/Derived Measures” theme encompasses metrics based on unclassified data, the “Relative Time” corresponds to the category “Proportion”, “Absolute Time” reflects the category “Duration”, and “Frequency” and “Point in Time” retained the same terminology. They also align well with the FITT framework used to describe PA dimensions, while expanding these concepts, as proposed by others before [135, 136].

Through this process, we observed that visualisation choices are more effectively guided by groups of metrics, the underlying data characteristics, and the research questions, than by individual metrics. For instance, output metrics that are used to describe frequency, regardless of measurement unit or specific movement behaviour represented, are visualised in similar ways, e.g. event plot. The detailed grouping, description and use of the visualisations included in the framework can be found in the Supplementary Material 5.

**Fig. 3:**
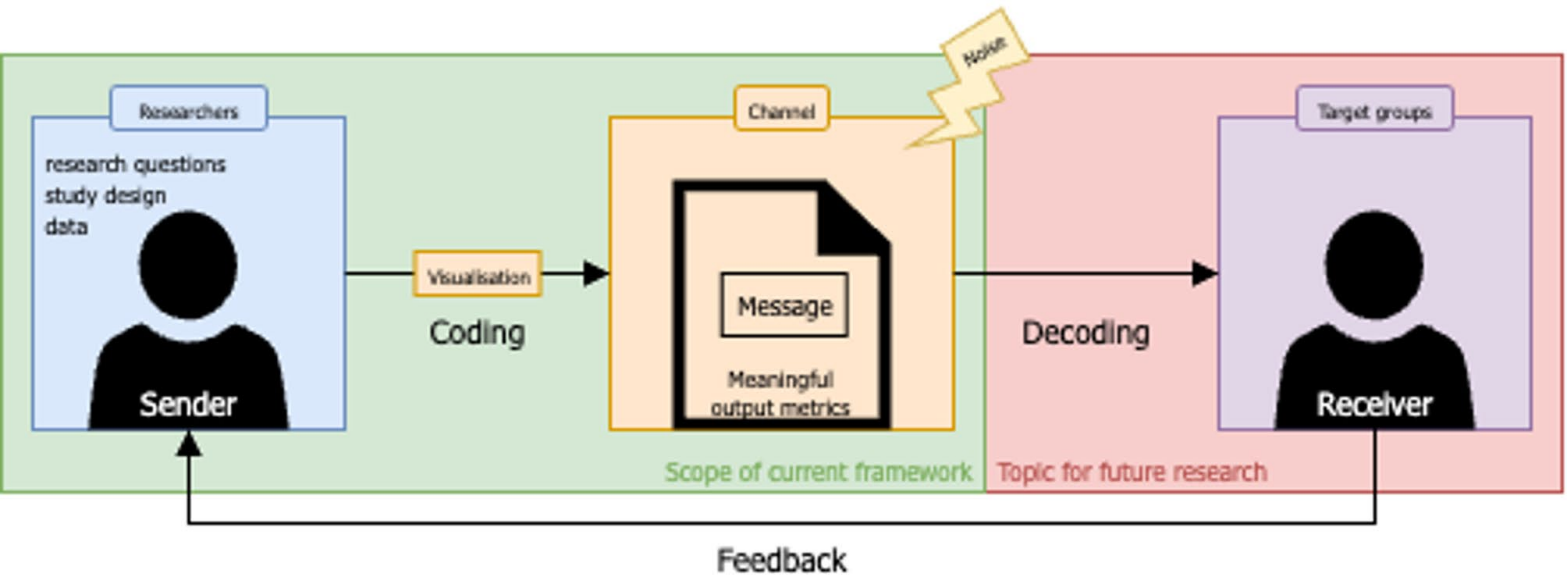
Rationale for developing a framework for human 24/7 movement behaviour visualisations based on the sender-receiver model

The resulting conceptual framework was the product of the above-described iterative process that aggregated the literature review results, the non-systematic searches for the visualisations, relevant publications, and the authors’ expertise. By applying the sender-receiver model [23] to this context, the researcher acts as the sender, responsible for clearly defining the research questions and identifying the target audiences (receivers). The visualisations function as the coding strategy, which involves transforming raw data into a visual format that aims to clarify and highlight the intended message, while the data themselves are the core content, or message, represented by the output metrics. The medium or channel through which this encoded message is transmitted includes papers and conference presentations. Finally, the target group is responsible for decoding the message, which involves interpreting the data visualisation. To facilitate effective decoding, it is crucial to minimise unnecessary noise in the visualisation and tailor it to the audience’s needs. We used the sender–receiver model primarily as a theoretical lens to highlight that visualisations are not only tools for data analysis but also part of a broader communication process. By focusing on clear and effective communication through data visualisation, researchers can increase the chances that their findings reach the intended audience and have a broader impact. Figure 3 illustrates the conceptual rationale that guided the framework’s development, embedding our findings within the sender-receiver model for effective communication.

We highlight that the researcher’s context includes considering which domains in 24/7 movement behaviour data to study, either in isolation or in combination, the study design, and other methodological and analytical choices that generate the output metrics. The decision-making points included in the framework, also part of the researcher’s context, include identifying the type of data (unclassified or classified), the metrics that represent the message to be transmitted, and the corresponding research question(s) they address, here translated into the framework categories. The coding process of choosing the adequate visualisations is a result of these choices, but also goes through traditional data visualisation criteria, such as considering if you want to visualise one variable alone, two, three, or more, if the data is grouped (e.g. boys and girls data) or ungrouped, among others [137].

**Fig. 4:**
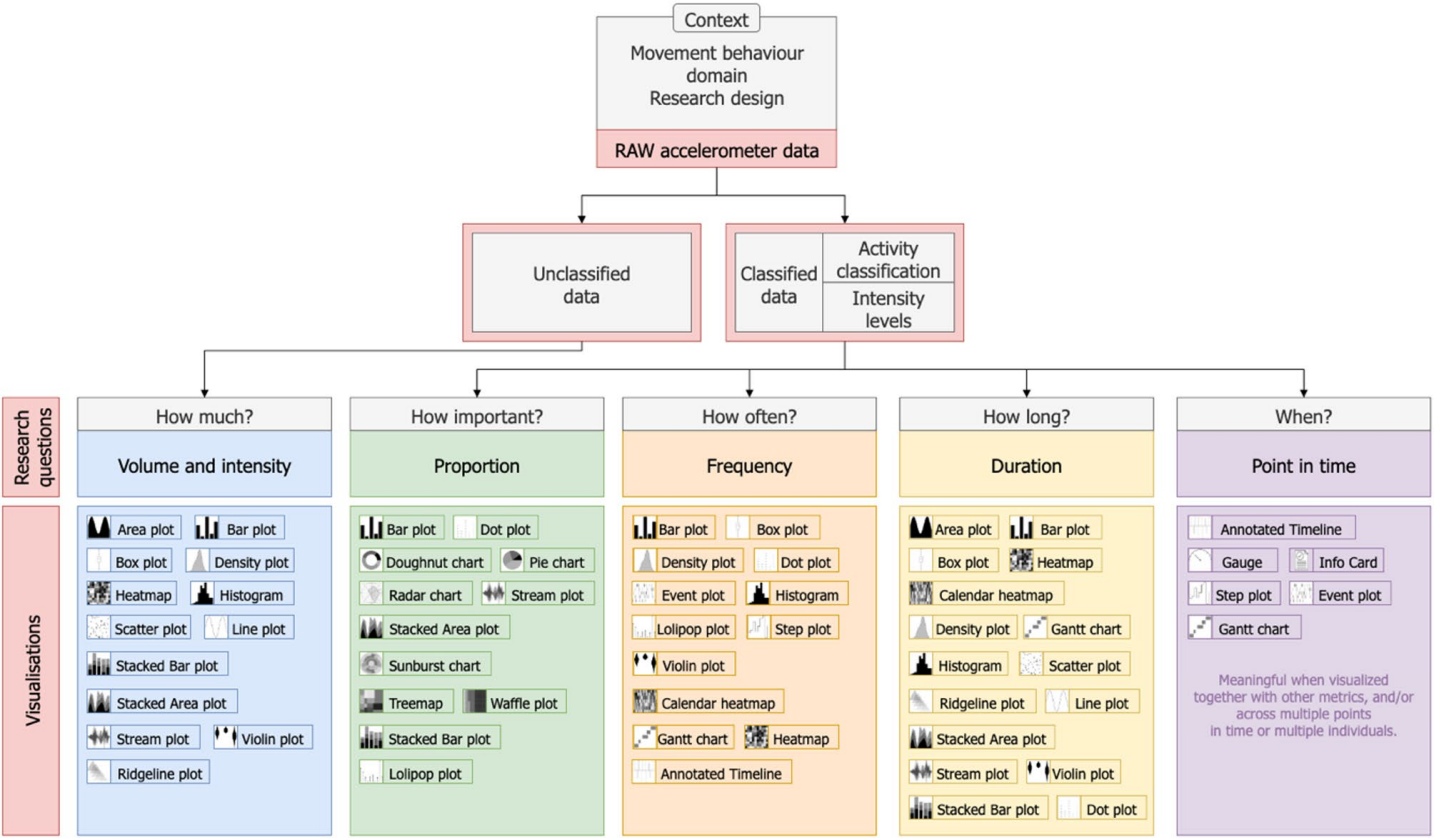
Framework for the selection of visualisations of metrics based on accelerometer data to communicate 24/7 movement behaviour

The proposed framework, presented in Fig. 4, does not provide prescriptive recommendations, but offers a systematic and practical guide for organising and selecting 24/7 movement behaviour metrics and their corresponding visualisations.

**Fig. 5:**
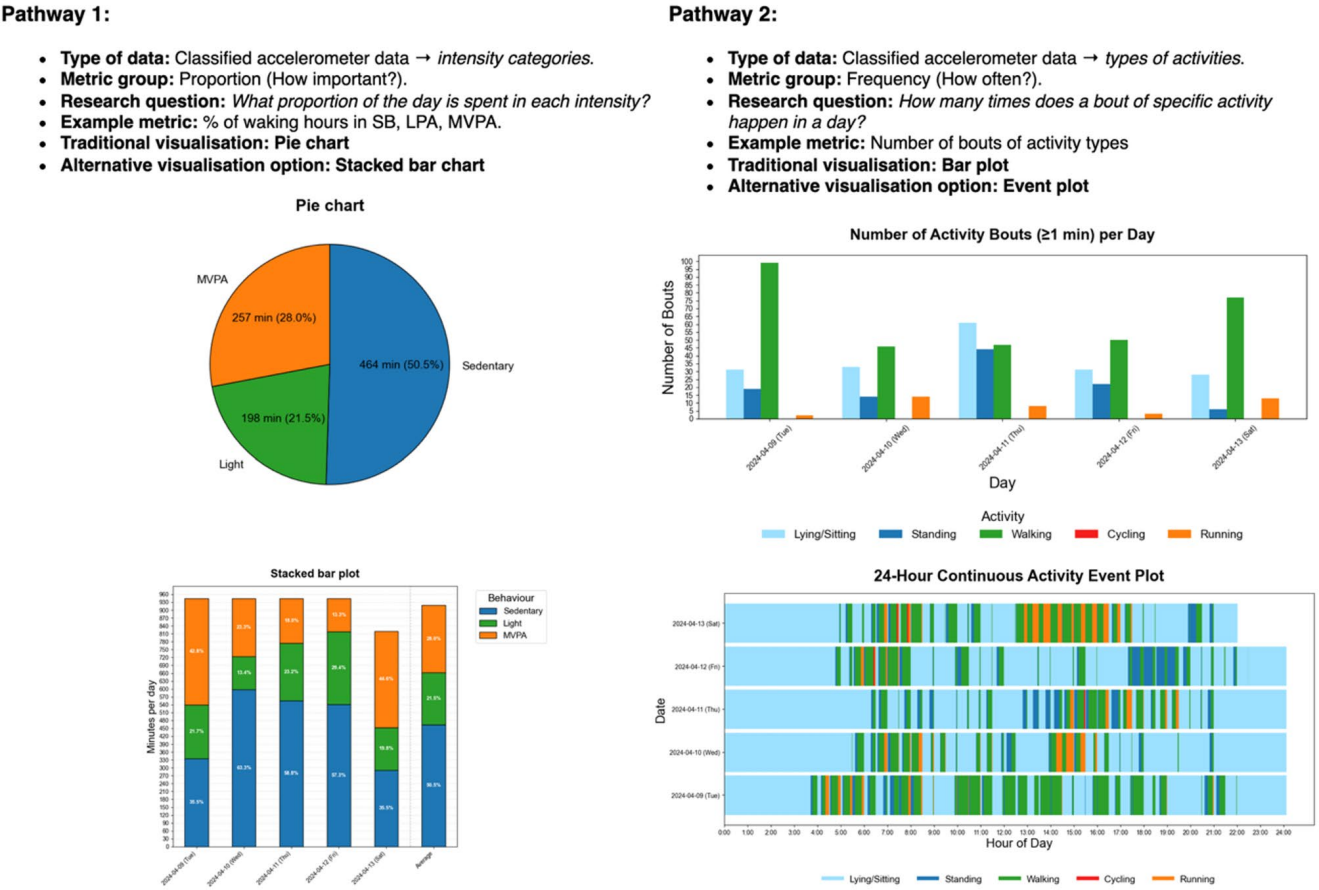
Illustration of the use of the framework for the selection of visualisations of metrics based on accelerometer data to communicate 24/7 movement behaviour. Pathway 1 contrasts a simple overview of proportions shown in the Pie Chart with a Stacked Bar Plot, which shows daily variation, temporal patterns, and allows for comparison between averages and individual days. Pathway 2 show the visualisation of different activity bouts count through a Bar Plot, which can only provide the number of bouts, versus an Event Plot, which also shows individual bouts’ durations and temporal patterns

Figure 5 presents two example pathways a researcher might follow within the framework, each illustrated with both a traditional and a less commonly used visualisation. The latter may offer greater insight depending on the intended message. These examples are meant to demonstrate how the framework can be applied, rather than prescribe specific visualisations. Further work on design features and interpretability is still needed before we can identify the most effective options for the scenarios shown.

## Discussion

The present study highlights the diversity of 24/7 movement behaviour metrics derived from accelerometers identified across 93 reviews, published between 2012 and 2024, including a total of 5667 original studies. The identification of 134 distinct metrics and the diverse visual representations we mapped to them underscore the considerable heterogeneity in how movement behaviour data are communicated. Currently, the lack of structured guidance in the visual communication of 24/7 movement behaviour data may reflect on the difficulty in selecting visualisation techniques considering data characteristics or communication goals. To address this gap, we propose a conceptual framework to guide researchers to the most suitable visualisations for their message, based on different movement behaviour components, types of metrics, and the key features of 24/7 movement behaviour.

To put these findings into context, it is important to consider that it is increasingly evident that the integrated distribution of 24/7 movement behaviours may be more relevant for health outcomes than PA alone [6, 12, 138]. This broader perspective motivated our goal of compiling accelerometer-derived metrics describing PA, SB and sleep. Consistent with prior work [139], our umbrella review revealed a clear need for establishing a common taxonomy to ensure consistent language in movement behaviour research. Although we compiled frequently used metrics in the field, the variability in nomenclature and lack of consensus hinders efforts to create a unified terminology. Adopting a common taxonomy, such as that proposed by Chastin et al. [140], would standardise summary and output metrics, thereby facilitating consistent visualisation approaches. In addition to adopting a common taxonomy, harmonisation of accelerometer data, as exemplified by initiatives like the ProPASS consortium [141], is critical for ensuring that metrics, and consequently visualisations of 24/7 movement behaviours, are more consistent. This, in turn, would enhance comparability across studies and potentially improve comprehension of results.

Although work has been done to improve accuracy and usability [142–144], reliability [145], applicability and interpretability [146], and study association with health outcomes [147] of accelerometer-derived 24/7 movement behaviour metrics, their visual communication remains largely overlooked. Across the 93 reviews included in our umbrella review, we found no explicit discussion of how visualisations were used, evaluated, or selected to communicate movement behaviour metrics. This highlights the absence of a structured approach to visualising these data, such as the one proposed in this study, to support effective and transparent communication of 24/7 movement behaviour data.

Data science leverages visualisation and visual analytics for interactive exploration, leading to deeper understanding, uncovering hidden patterns, and guiding informed decision-making [148]. While related work has been done on the visualisation of movement for Geographic Information Science [149, 150] and animal behaviour analysis [151], which focus on movement trajectories and GPS data, the visualisation of 24/7 movement behaviour metrics remains fragmented. Some studies provide examples of case-specific or general domain visualisation methods for accelerometer data [152–155], which were not captured in our metric-focused searches, but represent individual efforts to tailor visualisations to the data they represent. This corroborates how current practices tend to be case-specific, influenced by research team capabilities and preferences rather than guided by systematic reasoning [156], suggesting that existing visualisation methods may not be optimally utilised. The situation gets especially complicated with continuous and time series data, since interpretation and critical analysis of these visualisations is harder in some of the most often used representations in the field [156]. Additionally, our attempts to systematically identify peer-reviewed publications on visualisation methods for accelerometer metrics yielded no useful results, suggesting that the importance of effective visual communication in this field is underappreciated.

This review represents a first step towards broadening the current approach in movement behaviour research, emphasising data visualisation and communication throughout the study design process. Effective visualisation is crucial for translating findings into accessible messages for practical settings such as healthcare [157] and policymaking [158].

Presenting our findings visually as a framework aligns with this perspective shift and enhances accessibility to our findings. It structures complex information clearly in a logical flow and categorises the metrics, allowing for conceptual coherency across studies, while breaking the decision-making process into manageable steps. Our framework may promote consistency in visualisation selection, supporting more consistent knowledge dissemination and application. Broader use of diverse visualisation types holds the potential to improve understanding, engagement, and communication of 24/7 movement behaviour data [159, 160]. Expanding beyond traditional bar and line charts can reveal nuances and patterns otherwise obscured, which may ultimately enhance the impact of research findings [161, 162].

Moreover, strategies for clearer communication of evidence-based PA guidelines are necessary for an effective positive impact on health [163], which must be tailored to different audiences. The Physical Activity Messaging Framework created by Williamson et al. [139] underscores the need for evidence-based, audience-specific messaging and highlights the importance of standardised terminologies. These works support the emphasis our framework places on visualisation as a vital component of communication of study results. For instance, general audiences and non-specialists in movement behaviour may not fully understand terms like MVPA, and improved visualisations of 24/7 movement behaviour metrics could help convey the intended messages of current PA guidelines more effectively. Another important aspect is the representation of uncertainty or variability in grouped data, as presenting only point estimates may mislead audiences without statistical expertise; incorporating measures to show variability can provide a more accurate understanding of the results [164]. This need for clear communication is further highlighted by the WHO’s latest report on trends in prevalence of insufficient PA among adults, which showed a global increase in insufficient PA since 2000, with disparities by sex, age, and geography [165]. Effective visualisation of metrics and consequent communication of guidelines is crucial to support PA promotion efforts and address these public health challenges and disparities [158].

To our knowledge, this is the first work to both gather the metrics commonly used to describe 24/7 movement behaviour data from accelerometers and develop a tool to guide the choice of visualisations for these metrics. We believe that the framework developed represents an important step in movement behaviour research, particularly in enhancing communication and visualisation of findings. Grounding the framework in the sender-receiver communication model promotes awareness of effective communication processes. We recommend future development of the framework, tailoring the visualisations to diverse target audiences. By being aware of their targeted receivers, and of their own perspectives as senders of the message, researchers hopefully can select visualisations that reduce confusion and effectively convey key messages.

It is important to note that this paper combines different research traditions. While the umbrella review of accelerometer-derived metrics represents the systematic component, the scarcity of evidence on visualisation required a shift toward a framework development approach [166, 167]. In this phase, the literature review was used as input for generating new conceptual knowledge, an unorthodox but necessary step to establish a theoretical basis for treating data visualisation as a communication rather than analytical tool in 24/7 movement behaviour research.

We acknowledge that this work has limitations. Firstly, no protocol was pre-registered for this review, and we acknowledge that pre-registration improves transparency, reproducibility, and methodological rigor of systematic literature reviews. Secondly, the framework requires ongoing refining, including the incorporation of newer metrics and visualisation methods as the fields evolve. Furthermore, since it includes reviews published up to 2024, and the papers in these reviews were published before that, the metrics compiled may not represent the most recent body of literature. Additionally, our visualisation search strategy was focused on metric-specific representations considering the visualisation tools as part of the investigation purpose, which may have been too narrow to capture the full range of visualisation practices used in accelerometer research. Finally, the framework does not make specific recommendations but serves as a guide to support researchers in selecting suitable visualisations aligned with their research design.

Another important point to be considered is that the framework does not yet incorporate feedback from users. We are aware that visualisation of results impacts the communication and understanding of the message, and that it needs to be critically considered through the whole research design process [19, 168]. While the visualisations in our framework align with data types and research questions, it remains unclear how well visualisations communicate results, or what types of visualisations are preferred by diverse audiences. Future research should investigate how different target groups perceive these visualisations, to refine selection processes and enhance decision-making efficiency. An ideal future iteration of the framework would extend to include guidance on visualisation choices tailored to specific audiences, thereby maximising communication effectiveness. Ways of tailoring visualisations to different audiences could include co-design with end users, empirical evaluation of comprehension and usability, or iterative testing of visualisation prototypes.

Nevertheless, we hope that our proposed framework guides researchers’ decision-making process towards adequate and more diverse visualisation possibilities, as well as generates a body of literature with more standardised visualisations for this field of research.

## Conclusions

The present study highlights the diversity of accelerometer-derived 24/7 human movement behaviour metrics, which contrasts with the lack of studies regarding visualisation of these metrics. We developed a framework that aims to guide researchers in selecting appropriate visualisations for their context, thereby contributing to a systematic approach to data visualisation. The framework may support researchers in improving the clarity, impact, and accessibility of their findings. Future research is needed to gather information on how the visualisations presented here are perceived by diverse audiences.

## Supplementary Information

Below is the link to the electronic supplementary material.

Supplementary Material 1: Preferred Reporting Items for Systematic reviews and Meta-Analyses extension for Scoping Reviews (PRISMA-ScR) Checklist

Supplementary Material 2: Umbrella review data extraction table. It summarises the data extracted from the 93 reviews on title, first author, review publication year, journal, DOI, type of review, range of publication year of included papers, study design of included papers, number of papers included, number of papers which used accelerometers as an assessment tool, population characteristics (age and disease status), exposure(s), outcome(s), if visualisation tools were discussed or mentioned, and the accelerometer-derived output metrics used to describe 24/7 human movement behaviours

Supplementary Material 3: Details on the tentative systematic search for the visualisation tools, for transparency of the process

Supplementary Material 4: Data analysis documentation. It includes details about the process of coding and thematic data analysis, better describes the output metrics compiled, including the units of measurement and time granularity (e.g. time in seconds, minutes hours, days, etc.), and contains information on which review cited each metric, how many reviews cited each metric, and the grouping of metrics

Supplementary Material 5: Detailed description of the visualisations. It includes information on the name of the visualisation, general use cases, category of the framework that contains the metrics it can be used as visualisation tool, and the reasons why they are a good fit for the metrics in these categories

## Data Availability

The data gathered for this umbrella review is described in the Supplementary Material 2.

## Abbreviations

AI0: Activity Intensity
ENMO: Euclidean Norm Minus One
FITT: Frequency, Intensity, Time and Type
GPAQ: Global Physical Activity Questionnaire
PAQI: International Physical Activity Questionnaire
LABDA: Learning Network for Advanced Behavioural Data Analysis
LPA: Light intensity physical activity
MIMS-unit: Monitor-Independent Movement Summary unit
MPA: Moderate Physical Activity
MVPA: Moderate-to- Vigorous Physical Activity
PA: Physical Activity
SB: Sedentary Behaviour
VPA: Vigorous Physical Activity
WHO: World Health Organization
