# Supplementary material for: Visualising accelerometer-based 24/7 human movement behaviour data: an umbrella review and framework development from the LABDA project
Source: J Act Sedentary Sleep Behav. 2025 Nov 18;4:19. doi: 10.1186/s44167-025-00088-6 (PMC12625468; doi:10.1186/s44167-025-00088-6)
Supplement: Supplementary file 1 — Supplementary Material 1: Preferred Reporting Items for Systematic reviews and Meta-Analyses extension for Scoping Reviews (PRISMA-ScR) Checklist [file 44167_2025_88_MOESM1_ESM.docx]

**Additional File 4.** Detailed description of the visualisations.

| **Visualisation** | **Use Case** | **Framework Category** | **Why It's a Good Fit** |
| --- | --- | --- | --- |
| **Annotated timeline** | Key events over time | Point in Time, Frequency | Adds context to when specific transitions or thresholds occurred |
| **Area plot** | Change in volume (amount) over time | Duration, Volume/Intensity | Helps visualise cumulative or layered data over time |
| **Bar plot** | Comparisons of values across categories | Frequency, Volume/Intensity, Duration | Flexible and interpretable; good for both discrete and binned data |
| **Box plot** | Summary statistics + variability | Volume/Intensity, Duration | Highlights medians, IQRs, and outliers effectively |
| **Calendar heatmap** | Daily/weekly patterns | Frequency, Duration | Useful for time-of-day or date-level insights across long durations |
| **Density plot** | Smooth distribution estimate | Volume/Intensity, Duration | Highlights trends and patterns; good for comparing groups |
| **Dot plot** | Small counts or binary comparisons | Frequency, Proportions, Volume | Minimalist and effective; also useful for multiple groups |
| **Doughnut chart** | Variation of pie chart with center space | Proportions | Offers extra annotation space in center, still limited to few categories |
| **Event (Raster) plot** | Individual events across time/people | Frequency, Point in Time | Highlights timing and repetition of discrete events |
| **Gantt chart** | Duration and sequence of events | Frequency, Duration, Point in Time | Best for visualising timing, order, and overlap of behaviours (e.g., sleep, activity bouts) |
| **Gauge** | One-off snapshot of a single value | Point in Time | Useful only for summary metrics per person/period; not suitable for trend analysis |
| **Heatmap** | Patterns in matrix/time or intensity | Volume and Intensity, Duration, Frequency | Highlights concentration or intensity across dimensions |
| **Histogram** | Distributions of numeric values | Volume/Intensity, Duration | Shows data distribution clearly; best for continuous data |
| **Info box or card** | Summary statistics or one key value | Point in Time, Any (summary level) | Supports dashboards or infographic-style outputs; not for raw data visualisation |
| **Line plot** | Time series trend | Duration, Volume/Intensity | Ideal for continuous data over time |
| **Lollipop plot** | Minimalist bar chart | Frequency, Proportions | Emphasizes values clearly with less clutter |
| **Pie chart** | Simple parts of a whole | Proportions | Best with 3–4 categories; limited readability |
| **Radar chart** | Multivariate categorical comparison | Proportions | Useful to compare different "profiles" (e.g., activity across categories) |
| **Ridgeline plot** | Distribution across multiple categories | Volume/Intensity, Duration | Shows multiple distributions simultaneously, especially over time or groups |
| **Scatter plot** | Relationship between two variables | Volume/Intensity | Great for exploring correlation or variation in continuous metrics |
| **Stacked area plot** | Compare parts of a whole over time | Volume and Intensity, Duration, Proportions | Shows how components evolve together over time |
| **Stacked bar plot** | Composition of values by group | Volume and Intensity, Duration, Proportions | Compares categorical parts of a whole clearly |
| **Step plot** | Sudden changes (e.g. transitions) | Frequency, Point in Time | Ideal for visualising state changes (e.g., sleep/wake, posture shifts) |
| **Stream plot** | Flow of multiple values over time | Duration, Proportions | Aesthetic variant of stacked area plot with fluid shapes |
| **Sunburst chart** | Multilevel proportions | Proportions | Great for hierarchical part-to-whole visualisations |
| **Treemap** | Hierarchical proportion representation | Proportions | Space-efficient way to show parts of a whole, especially with many categories |
| **Violin plot** | Distribution + summary statistics | Volume/Intensity, Duration | Combines box plot with density estimation for richer context |
| **Waffle plot** | Grid-based proportion representation | Proportions | More visual and countable than pie charts; clearer proportions |
