# Supplementary material for: Visualising accelerometer-based 24/7 human movement behaviour data: an umbrella review and framework development from the LABDA project
Source: J Act Sedentary Sleep Behav. 2025 Nov 18;4:19. doi: 10.1186/s44167-025-00088-6 (PMC12625468; doi:10.1186/s44167-025-00088-6)
Supplement: Supplementary file 3 — Supplementary Material 3: Details on the tentative systematic search for the visualisation tools, for transparency of the process [file 44167_2025_88_MOESM3_ESM.docx]

**Additional File 3**

Tentative search blocks for the systematic PubMed searches for visualisations of metrics and groups of metrics, performed in May 2025. Only papers in English were retrieved, and no other filter was applied to this search.

1. Search for visualisations for specific metrics (one search for each of the 132 metrics) as variations of the following search block:

“(metric of interest) AND (“movement behavio*” OR “activity behavio*” OR “physical activity” OR PA OR “sedentary behaviour” OR sedentary OR SB OR sleep) AND (“data visuali*” OR visuali*)”, for instance “(“accelerometer counts” OR counts) AND (“movement behavio*” OR “activity behavio*” OR “physical activity” OR PA OR “sedentary behaviour” OR sedentary OR SB OR sleep) AND (“data visuali*” OR visuali*)”.

1. Search for visualisations corresponding to the identified metric categories (one search for each of the five categories):

“(group of metrics of interest) AND (“movement behavio*” OR “activity behavio*” OR “physical activity” OR PA OR “sedentary behaviour” OR sedentary OR SB OR sleep) AND (“data visuali*” OR visuali*)”, for instance “(frequency) AND (“movement behavio*” OR “activity behavio*” OR “physical activity” OR PA OR “sedentary behaviour” OR sedentary OR SB OR sleep) AND (“data visuali*” OR visuali*)”.
